# Investigation of the First Case of Dengue Virus Infection Acquired in Western Australia in Seven Decades: Evidence of Importation of Infected Mosquitoes?

**DOI:** 10.1371/journal.pntd.0004114

**Published:** 2015-09-25

**Authors:** Michael D. A. Lindsay, Andrew Jardine, Carolien Giele, Paul Armstrong, Suzi McCarthy, Amanda Whittle, Naru Pal, Heather Lyttle, Sue Harrington, Jay Nicholson, David Smith

**Affiliations:** 1 Public Health Division, Department of Health, Perth, Western Australia, Australia; 2 PathWest Laboratory Medicine WA, Department of Health, Perth, Western Australia, Australia; 3 School of Pathology and Laboratory Medicine, Faculty of Medicine, Dentistry and Health Sciences, University of Western Australia, Perth, Western Australia, Australia; 4 South West Population Health Unit, Department of Health, Bunbury, Western Australia, Australia; 5 Pilbara Population Health Unit, Department of Health, Port Hedland, Western Australia, Australia; Duke-NUS, SINGAPORE

## Abstract

In October 2013, a locally-acquired case of dengue virus (DENV) infection was reported in Western Australia (WA) where local dengue transmission has not occurred for over 70 years. Laboratory testing confirmed recent DENV infection and the case demonstrated a clinically compatible illness. The infection was most likely acquired in the Pilbara region in the northwest of WA. Follow up investigations did not detect any other locally-acquired dengue cases or any known dengue vector species in the local region, despite intensive adult and larval mosquito surveillance, both immediately after the case was notified in October 2013 and after the start of the wet season in January 2014. The mechanism of infection with DENV in this case cannot be confirmed. However, it most likely followed a bite from a single infected mosquito vector that was transiently introduced into the Pilbara region but failed to establish a local breeding population. This case highlights the public health importance of maintaining surveillance efforts to ensure that any incursions of dengue vectors into WA are promptly identified and do not become established, particularly given the large numbers of viraemic dengue fever cases imported into WA by travellers returning from dengue-endemic regions.

## Author Summary

Dengue fever transmission in Western Australia ceased in the 1940s and there are currently no known dengue vector species present. Despite this, a locally acquired of dengue fever was reported in 2013 with the most likely location of exposure the township of Point Samson in Pilbara region in the north-west of the State. A comprehensive follow-up investigation was undertaken and while the dengue case was confirmed, no other cases were identified and not exotic dengue vector mosquitoes were detected around the location of exposure. The exact mechanism for the locally acquired infection could not be determined but the most likely explanation is that a dengue infected mosquito was transported into the region, fed on the patient, but did not survive to lay eggs and establish a local breeding population. This case highlights the potential risk of the re-emergence of dengue fever in regions of Australia outside where ongoing transmission is currently limited to in northern Queensland, particularly given the large number of imported dengue cases that occur among travellers returning from dengue endemic regions.

## Introduction

Dengue fever is caused by a mosquito-borne flavivirus, dengue virus (DENV), and is responsible for a significant disease burden globally with an estimated 390 million cases of dengue fever per annum, of which 96 million result in symptomatic disease [1]. The incubation period is 3 to 14 days [2], and clinical manifestations range from mild symptoms, typically including fever, headache, rash, myalgia and arthralgia, to life-threatening haemorrhagic fever and severe shock [3]. In recent decades there has been a resurgence of dengue fever in many tropical and sub-tropical regions following the re-introduction of the primary mosquito vector species *Aedes (Stegomyia) aegypti (L*.*)* [4].

Transmission of DENV in Australia is currently restricted to urban areas of north Queensland and the Torres Strait where *Ae*. *aegypti* remains well established and outbreaks regularly occur following importations of dengue viruses by returning travellers infected overseas [5–7]. Recent incursions of *Ae*. *aegypti* into towns in the Northern Territory have occurred, but the vectors were eliminated without any dengue activity being detected [8–10]. In WA, outbreaks of dengue were reported in northern parts of the state up until the mid-1940s *[6]* and *Ae*. *aegypti* was recorded as far south as Harvey, approximately 150km south of the capital city, Perth, in the southwest of the State up until the 1950s [6]. However, the distribution of this species receded dramatically following changes in water storage practices and the introduction of scheme water supply, and disappeared completely by the end of the 1960s [6]. Other potential vectors of dengue, such as *Aedes (Stegomyia) albopictus* (Skuse), are not present in WA and no native mosquito species are known to have the potential to transmit DENV.

Dengue fever is a notifiable disease in WA, meaning that doctors and laboratories are legally compelled to report cases. In WA, public health units follow up all dengue fever cases to determine where the infections were acquired, if the notifying doctors have not provided the information. The vast majority of cases have been acquired overseas and, in the past five years, these have increased to several hundred cases annually, reflecting both the increase in Western Australians travelling to, and increasing dengue activity in nearby dengue-endemic countries, particularly Bali in Indonesia [11]. The Commonwealth Department of Agriculture also conducts routine surveillance to monitor incursions of exotic mosquitoes in areas surrounding international air and sea ports in WA.

In early October 2013, the Department of Health WA was notified of a laboratory-confirmed case of dengue in a male who had not travelled outside of WA in over a decade. This was the first report of a locally acquired case of dengue fever in the state for more than 70 years.

## Methods

### Case investigation

The case was interviewed to establish his symptomatology, date of onset and travel history in the two weeks prior to symptom onset to determine the most likely location of exposure. The Western Australian Notifiable Infectious Diseases Database (WANIDD) was examined to determine if any other locally-acquired cases of dengue had occurred in the area.

### Laboratory testing

A serum sample was collected from the patient on 1 October 2013 and referred for confirmation to PathWest Laboratory Medicine WA (PathWest), the state reference laboratory, for testing of dengue antibody titre by flavivirus haemagglutination inhibition (HI), dengue IgM by SD Dengue IgM Capture ELISA and dengue NS1 antigen by Bio-Rad Platelia Dengue NS1 Ag. The dengue serotype was also determined using in-house, type-specific, reverse transcriptase real-time double PCR. In addition, virus isolation was attempted in African green monkey kidney epithelial cells (Vero cells). A second sample collected 6 days later was retested using the same methods.

In WA, dengue virus testing is not routinely requested for patients presenting with a dengue-like illness unless they have travelled overseas or to northern Queensland. Therefore, in order to exclude other possible locally-acquired cases, all blood samples sent to PathWest laboratories in the Pilbara requesting routine arbovirus testing between 1 July 2014 and 13 October 2013 were re-tested for dengue IgM and NS1 antigen.

### Environmental investigation

**Fig 1:**
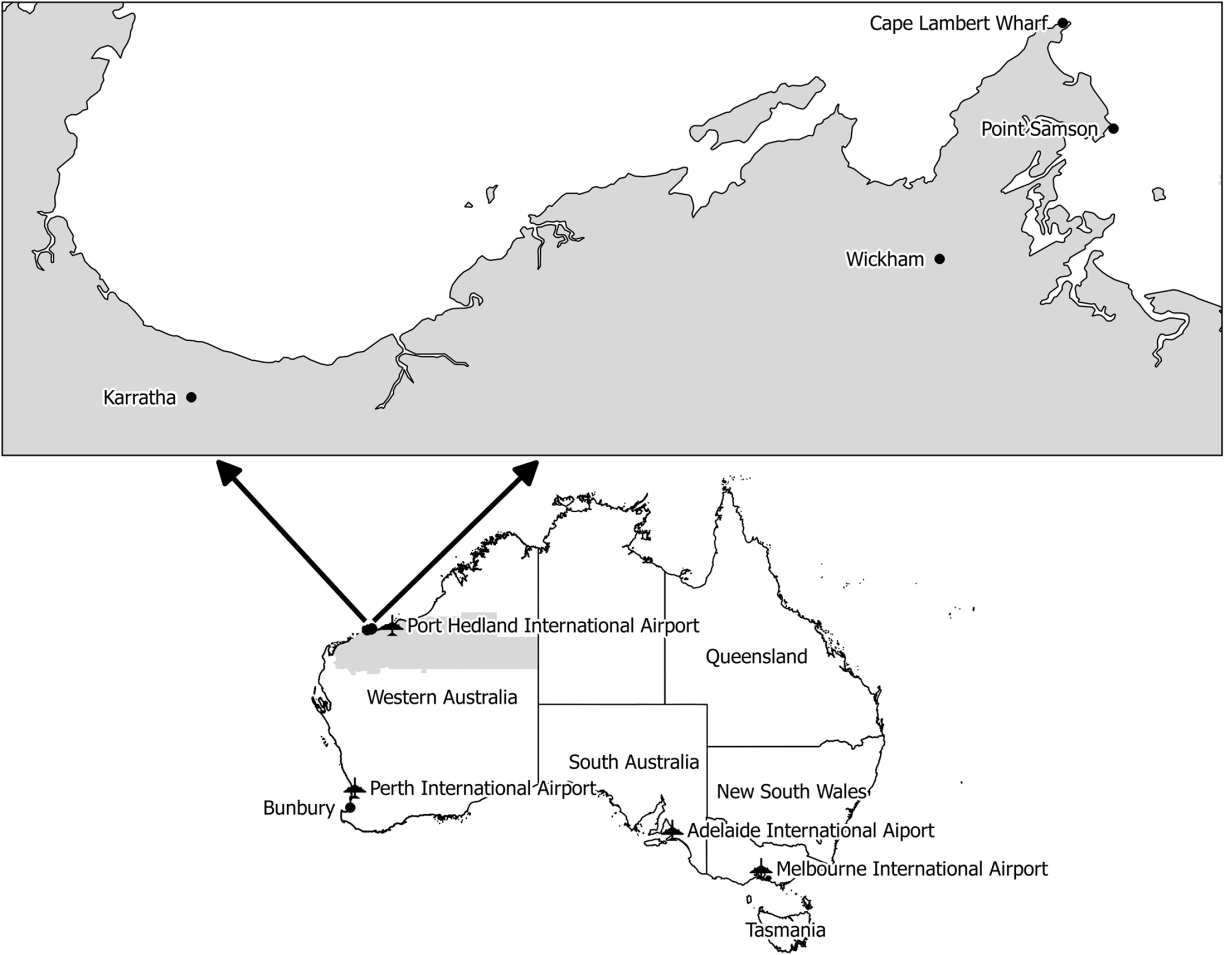
Map indicating Point Samson and other relevant locations. The Pilbara region is indicated by grey shading.

Intensive mosquito surveillance was carried out at the likely locations of exposure (Point Samson and Wickham townships in the Pilbara region in north-west WA (Fig 1)) between 17 and 25 October 2013. Adult mosquitoes were collected using carbon dioxide-baited encephalitis virus surveillance traps (EVS/CO_2_ traps) [12] modified to suit local environmental conditions, Biogents (BG) sentinel traps [13], and sticky ovitraps [14]. Extensive larval surveys of potential container breeding habitats were also undertaken.

A second mosquito survey of Point Samson, Wickham and Cape Lambert wharf (Fig 1) was undertaken after the start of the wet season in January 2014 when desiccation-resistant eggs of container-breeding exotic mosquito species would be expected to hatch.

## Results

### Case investigation

The case resided in a caravan park in the small coastal town of Point Samson in the Pilbara region of WA, located approximately 1500km north-east of Perth (Fig 1), and frequently travelled to Wickham, 10 km south-west of Port Samson. Both Point Samson and Wickham towns service mining operations in the region and Port Walcott (Cape Lambert wharf), a major iron ore exporting port located 3km north-west of Point Samson (Fig 1) where international cargo vessels dock.

The case reported onset of symptoms typical of dengue fever, including fever, headache, lethargy and rash, on 24 September 2013 while in Bunbury in the southwest of WA, two days after departing Point Samson (Fig 1). He denied having travelled outside WA for many years, which was confirmed by the Commonwealth Department of Customs and Immigration, and had not been in the vicinity of any international airports or seaports during the two weeks prior to onset of symptoms with the exception of Port Walcott, which he was unable to recall specifically if he had visited or been bitten by mosquitoes there during the potential exposure period. He also reported wearing heavy duty clothing with long sleeves for work duties. However, the case did recall being bitten by mosquitoes at his residence in Point Samson during this period. Therefore, the most likely place of exposure was considered to be Point Samson, followed by Wickham where he travelled frequently for work, or Port Walcott where he may have also visited.

In 2013, prior to this locally acquired case, eight other cases of dengue fever were notified among Pilbara residents; all reported recent travel to dengue-endemic countries. Of these, the most recent case had been reported over six weeks earlier and resided in Karratha (50km west of Point Samson). None of these eight cases were residents of Point Samson or Wickham and the patient had not visited Karratha.

### Laboratory testing

Testing by the reference laboratory confirmed dengue virus infection in two serum samples taken six days apart. The results from the first serum sample tested was dengue IgM positive, dengue NS1 antigen positive, PCR positive for DENV-1 and had a high flavivirus HI antibody titre [1:640]. The second sample yielded the same results, with the exception of a negative NS1 antigen test. Cultures for dengue virus were unsuccessful and further subtyping of the virus was also not possible as a DNA sequence from the PCR product could not be obtained.

One hundred and sixteen serum samples from 115 people submitted for routine arbovirus testing by PathWest’s Pilbara laboratories between 1 July 2014 and 13 October 2013 were re-tested for dengue IgM and NS1 antigen. One sample tested positive for dengue NS1 antigen and IgM and two further samples tested positive for dengue IgM alone. Further investigations revealed all cases had acquired a dengue infection overseas in a dengue-endemic country.

### Environmental investigation

The climate in the region of Point Samson is generally hot and dry with a mean annual rainfall of approximately 300mm, almost two-thirds of which falls during the wet season between January and March.

No exotic mosquito species were detected during the intensive mosquito surveillance undertaken at Point Samson and Wickham. The dominant native species collected as adults were *Culex quinquefasciatus* and various species in the *Aedes (Macleaya*) subgenus which were also the only species collected as larvae. Small numbers of adult mosquitoes of the following species were also collected: *Cx*. *annulirostris*, *Cx*. *sitiens*, *Ae*. *vigilax*, *Ae*. *bancroftianus and Anopheles novaguinensis*. None of these are known or suspected dengue vector species. While properties within the area of the survey contained a large numbers of potential breeding receptacles, only six actual breeding sites were found. Overall, mosquito abundance at the time of the survey was extremely low. There had been no rainfall since 24 June 2013 so it is likely that mosquito abundance would also have been low during mid-September when this man was most likely to have acquired his dengue infection.

Further intensive adult and larval mosquito surveys conducted in January 2014 following rainfall associated with the wet season also did not detect any known dengue vector species.

Routine surveillance for exotic mosquitoes by the Commonwealth Department of Agriculture at international air and sea ports in WA did not detect the dengue vectors *Ae*. *aegypti* and *Ae*. a*lbopictus* in the months prior to this case (personal communication, Aaron Maxwell, Assistant Director–Operational Science Services, Department of Agriculture Western Australia).

### Public health intervention

The Department of Health issued a media statement on 15 October 2013 to warn the public of the possibility of local dengue virus transmission while the Pilbara Population Health Unit requested local doctors to test patients presenting with dengue-like symptoms. Other mosquito borne flaviviruses are relatively common in the Pilbara, so doctors were asked to include requests for dengue virus when testing for arboviral infections, whether or not the person had travelled overseas.

## Discussion

This paper reports the first case of dengue fever acquired in WA for the past 70 years. The case had not travelled outside WA for over a decade and laboratory test results confirmed a recently acquired DENV infection. Point Samson was the likely location of exposure as he recalled being bitten by mosquitoes there, but Wickham could not be ruled out as the case had also travelled there frequently for work during the potential exposure period, and neither could Port Walcott where he may have also visited. As northern WA has had epidemic dengue in the past, this case has renewed concern about the potential for re-establishment of dengue in the area.

The initial concern was that the DENV infection in this case may have been transmitted by an exotic dengue vector mosquito that had become established in the Pilbara region and been infected with DENV by feeding on a viraemic returned traveller. This is analogous to the ongoing situation in northern Queensland [5–7]. While the existence of an established local breeding population of dengue vector mosquitoes could not be completely excluded by this investigation, neither the initial nor follow-up mosquito surveys found any evidence to support this scenario. In addition, no other cases were identified in the retrospective laboratory testing of patient sera with suspected arbovirus infections, although the possibility that further cases occurred but were not tested cannot be completely excluded. Similarly, in the absence of any DENV activity in mosquitoes or humans in the Pilbara prior to this case, it is very unlikely that it was introduced into a local native species with previously unrecognised vector competence.

The most likely explanation for this locally acquired case is that a DENV-infected mosquito harbouring in luggage or in other items was transported into the region either by ship recently arrived from overseas, by road from northern Queensland, or via a direct international flight into Perth or Port Hedland International Airports (Fig 1) from dengue-endemic countries. The case may have been bitten by the infected mosquito while visiting Port Walcott or the mosquito could have been transported to Point Samson, where it escaped and fed on the patient, but did not survive to lay eggs and reproduce in the dry and inhospitable environment at that time of year. While the probability of such a scenario occurring is low, it certainly cannot be discounted entirely and is the best explanation based on the available evidence.

Transient introduction by air of an infected dengue vector was regarded as the most plausible cause of a locally-acquired dengue fever case in Darwin in the Northern Territory in 2010 [15]. Passive transportation of mosquitoes by both air and sea has also been demonstrated in previous studies in the south-east Asian region [16, 17] and this has been suggested as a mechanism for spread of dengue fever internationally [4]. Further evidence to support this scenario is provided by the detection of exotic mosquitoes, including *Ae*. *aegypti*, at Perth, Adelaide and Melbourne International Airports (Fig 1) in 2014. The most regular detections occurred at Perth International Airport, but there is no evidence to suggest that *Ae*. *aegypti* became established locally [18].

We cannot be certain about the mechanism for local infection with DENV in this case. However, intensive surveys of mosquito fauna in the region of likely exposure immediately after serological confirmation of the case and again following wet season rains, together with enhanced screening and surveillance for other dengue cases found no evidence of an ongoing risk of dengue infection in the region. This case, and recent detections of *Ae*. *aegypti* at several international airports in Australia, highlights the potential risk of the incursion and establishment of DENV vectors in areas of Australia outside northern Queensland. If such vectors become re-established, the risk of local dengue outbreaks occurring would be very high due to the large number of infected returning travellers [11].

The ongoing public health surveillance and rapid response measures described in relation to this case remain an important public health priority in view of the well documented history of the widespread occurrence of *Ae*. *aegypti* and outbreaks of dengue fever in WA.

## Supporting Information

S1 File Mosquito surveillance data collected during the environmental investigation. (XLSX)
